# Charge Distribution in Layered Lanthanide-Doped CuCr_0.99_Ln_0.01_S_2_ (Ln = Pr–Tb) Thermoelectric Materials

**DOI:** 10.3390/ma15248747

**Published:** 2022-12-08

**Authors:** Evgeniy V. Korotaev, Mikhail M. Syrokvashin, Irina Yu. Filatova, Aleksandr V. Sotnikov, Alexandr V. Kalinkin

**Affiliations:** 1 Nikolaev Institute of Inorganic Chemistry, Siberian Branch, Russian Academy of Sciences, 630090 Novosibirsk, Russia; 2 Boreskov Institute of Catalysis, Siberian Branch, Russian Academy of Sciences, 630090 Novosibirsk, Russia

**Keywords:** layered copper-chromium disulfide, XPS, lanthanides, Seebeck coefficient, thermoelectricity

## Abstract

The charge distribution study of metal atoms in CuCr_0.99_Ln_0.01_S_2_ (Ln = Pr–Tb) solid solutions was carried out using X-ray photoelectron spectroscopy (XPS). The analysis of the binding energy of S2p, Cu2p, Cr2p, Ln3d and Ln4d levels allows one to determine the oxidation state of atoms. Copper atoms were found to be monovalent. Chromium and lanthanide atoms were found to be in the trivalent state. Sulfur atoms were found to be in the divalent state. Cationic substitution was found to occur via an isovalent mechanism of Cr^3+^ to Ln^3+^. The obtained results were used for the interpretation of the Seebeck coefficient increase for CuCr_0.99_Ln_0.01_S_2_ solid solutions in contrast to the initial CuCrS_2_ matrix. The largest Seebeck coefficient values of 142 and 148 µV/K were observed at 500 K for CuCr_0.99_Sm_0.01_S_2_ and CuCr_0.99_Pr_0.01_S_2_, respectively. The obtained values are 1.4 times greater in comparison with those for the initial matrix (105 µV/K).

## 1. Introduction

One of the actual trends of materials science is the design and improvement of high-efficient thermoelectric materials. These materials could be applied in compact solid state semiconductor devices for energy harvesting and autonomous electrical systems [1]. Layered transition metal dichalcogenides MX_2_ (M—metal, X—chalcogen) are considered as promising thermoelectric materials. The dichalcogenide layers in MX_2_ can be considered as a planar polymer {MX_2_}_n_ molecule with large interlayer distance and, thereby, weak interlayer interactions. The interlayer space between the individual layers can be filled (intercalated) with atoms and ions of molecules. The intercalated objects can form conductivity channels through the interlayer space. Variation of the type and concentration of the intercalated objects allows one to alter the functional properties of layered dichalcogenides, such as anisotropic electronic and ionic conductivity, and thermoelectric and magnetic properties. The cationic substitution of metal atoms in MX_2_ layers was also found to affect the functional properties of layered dichalcogenides [2,3,4,5,6]. The combination of both thermoelectric properties and ionic conductivity of MX_2_ allowed these compounds to be considered as phonon-glass electron-crystal (PGEC) materials [7,8]. Phonon glasses usually have a low thermal conductivity, as a result of phonon scattering on mobile cations, and a high Seebeck coefficient value, due to the “fixed” electron-crystal matrix. The combination of these properties results in a high value of the thermoelectric figure of merit (ZT) of the layered transition metal dichalcogenides [7,8,9,10]. Thus, the prospects of direct modifications of functional properties allows one to consider MX_2_ as promising compounds for the design of new thermoelectric materials [2,4,5]. The layered intercalated copper-chromium dichalcogenide CuCrS_2_- and CuCrS_2_-based solid solutions are considered as promising functional materials due to the high values of ZT reported for the initial CuCrS_2_ matrix [11,12,13,14]. Note that the real sample structure (the absorbed molecules, the stoichiometric composition, the crystal structure, and defects on the sample surface) allows one to consider the polycrystalline particles of CuCrS_2_ as core-shell compounds [15,16,17,18]. The thermoelectric materials based on the core-shell compounds combine both low thermal conductivity and electrical resistivity due to the phonon and electron mean free path differences. Thus, one can conclude that reported high values of ZT for CuCrS_2_ could be mainly determined by the real sample structure. However, there is no theoretical limit of the ZT value. This fact stimulates scientists to design new thermoelectric materials and to optimize the parameters of the existing design [2,3,4,5,17]. The cationic substitution of metal atoms in the CuCrS_2_ matrix is an effective approach to improve the material’s functional properties [4,5,17,19]. For instance, the cationic substitution of chromium with iron atoms at the low-level doping concentration region of x ≤ 0.03 in CuCr_1-x_Fe_x_S_2_ allows one to consider these compounds as promising thermoelectric materials [4]. In recent years, one of the actual trends of material sciences has been the design of thermoelectric materials based on lanthanide chalcogenides [9,20,21,22,23]. The partially filled *f*-subshell of the lanthanides, especially for light lanthanides from La to Eu, allows one to affect the DOS (density of states) distribution near the Fermi-level region (in the valence band top and the conduction band bottom) [24,25]. The cationic substitution of chromium atoms in the CuCrS_2_ matrix with heavier lanthanide ions allows one to decrease the thermal conductivity due to the increase in phonon scattering. However, an increase in cationic concentration suppresses the thermoelectric properties of CuCr_1−x_M_x_S_2_ due to the metal–insulator transition (MIT) [4,26]. Hence, the solid solution’s low-level doping concentration is of special interest. Since solid solutions of CuCr_0.99_Ln_0.01_S_2_ doped with La and Ce have been studied previously [19,27], here we report the study of the extended range of lanthanide-doped CuCrS_2_-based solid solutions CuCr_0.99_Ln_0.01_S_2_ (Ln = Pr–Tb).

One of the main characteristics of thermoelectric materials is the Seebeck coefficient (*S*) value. The materials with high *S* values are considered for applications in thermoelectric generators and cooling systems [28,29]. The Seebeck coefficient for semiconductors can be expressed as a function of DOS and the charge carrier properties [30]:

(1) $$S=-\frac{k_{B}^{2}}{e}\frac{1}{n\mu_{n}+p\mu_{p}}\left\{\left[2-\frac{E_{F}}{kT}\right]n\mu_{n}-\left[2-\frac{E_{F}+E_{g}}{kT}\right]p\mu_{p}\right\}=-\frac{k}{e}\{\frac{\left[2+\ln\left(\frac{Nc}{n}\right)\right]n\mu_{n}-\left[2+\ln\left(\frac{Nv}{p}\right)\right]p\mu_{p}}{n\mu_{n}+p\mu_{p}}\},$$

where *k* is the Boltzmann constant; *e* is the electron charge; *n*, *p*, *µ_n_* and *µ_p_* are the concentration of electrons and holes and their mobility, respectively; *E_g_* is the band gap width; *E_F_* is the Fermi energy; and *N_c_* and *N_v_* are the effective DOS in the conduction band bottom and valence band top, respectively. According to Equation (1), the Seebeck coefficient is determined by both electronic structure features and charge carrier parameters. The charge carrier concentration is significantly affected by the dopant oxidation state [30]. Note that the most common oxidation state of lanthanides is +3. However, for some lanthanides, +2 (Sm, Eu) and +4 (Pr, Tb) oxidation states are also common [31]. Thus, the atom oxidation state study is of special interest for the interpretation of CuCr_0.99_Ln_0.01_S_2_ thermoelectric properties. X-ray photoelectron spectroscopy (XPS) is one of the most effective experimental techniques to study the oxidation state of atoms in molecules and solids [32]. Since the binding energy (BE) of the core level is characteristic for elements in a certain chemical environment, XPS allows one to determine the oxidation state of a certain atom. Here, we report the study of both the Seebeck coefficient temperature dependencies and the XPS analysis of charge distribution for a wide range of CuCrS_2_-based lanthanide-doped solid solutions CuCr_0.99_Ln_0.01_S_2_ (Ln = Pr–Tb). It should be noted that the Seebeck coefficient and XPS lines for the lanthanide-doped CuCrS_2_-based solid solutions CuCr_0.99_Ln_0.01_S_2_ (Ln = Pr–Tb) were measured for the first time.

## 2. Experimental

Powder samples of the CuCrS_2_ matrix and CuCr_0.99_Ln_0.01_S_2_ solid solutions were synthesized using commercial metal oxides CuO, Cr_2_O_3_ and Ln_2_O_3_ (Ln = Pr…Tb) with a purity of 99.99%. The gaseous products of thermal decomposition of ammonium rhodanide NH_4_CNS were used as sulfurizing agents [19,27].

The phase composition was analyzed by X-ray powder diffraction (XRD) using a non-monochromatic CuKα-radiation (λ = 1.5406 Å) on a Shimadzu XRD 7000S diffractometer. The unit cell parameters of CuCr_0.99_Ln_0.01_S_2_ were calculated using PowderCell 2.3 based on the full profile Rietveld refinement method [33].

XPS measurements were carried out using a SPECS spectrometer with a PHOIBOS-150 hemispherical electron energy analyzer. The copper, lanthanides and sulfur XPS lines were recorded with a non-monochromatic AlKα radiation source (hν = 1486.6 eV). The chromium XPS lines were recorded with a non-monochromatic MgKα radiation source (hν = 1253.6 eV). The samples were fixed on a sample holder using conductive, double-sided adhesive tape. During the measurements, the samples were held at room temperature in a vacuum of 10^−9^ Torr. The spectrometer energy scale was calibrated according to the metallic gold Au4f_7/2_ (84.0 eV) and copper Cu2p_3/2_ (932.6 eV) line positions. After the measurements, the BE scale in the corresponding energy regions was calibrated using the internal standard, using the C1s line energy position (284.8 eV) for carbon atoms in the near-surface layers of the samples studied [16,24]. The measured spectra were decomposed into individual components and the background was subtracted by the Shirley method in CasaXPS 2.3.15 [34]. The BE measurement accuracy was 0.2 eV.

The Seebeck coefficient temperature dependencies of CuCr_0.99_Ln_0.01_S_2_ (Ln = Pr–Tb) were measured in a rarefied 5 Torr helium atmosphere. The synthesized powder samples were compressed at 923 K in a vacuum (5 × 10^−5^ Torr) under a uniaxial pressure of 70 MPa for 2 h. The compressed ceramic samples were placed between two copper contact pads with built-in 100 W heaters. The temperature gradient of 5 K applied to the sample was controlled by a Thermodat-13K5 temperature controller. The thermoelectric power arising from the sample was recorded using a 6½ Keysight 34465A multimeter.

## 3. Results and Discussion

### 3.1. X-ray Diffraction (XRD)

**Figure 1:**
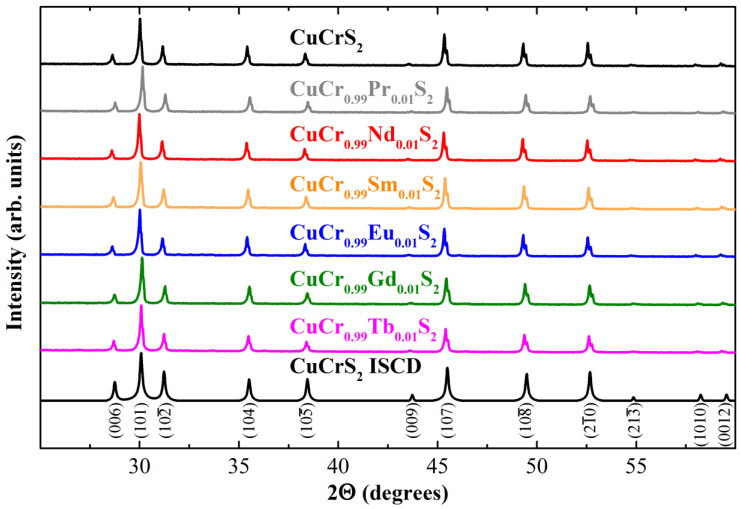
Powder diffraction patterns of the initial CuCrS_2_ matrix and CuCr_0.99_Ln_0.01_S_2_ (Ln = Pr, Nd, Sm, Eu, Gd, Tb) solid solutions.

The XRD patterns of the samples studied are shown in Figure 1. The diffraction peaks observed for CuCr_0.99_Ln_0.01_S_2_ solid solutions correspond to the initial CuCrS_2_ matrix and a *R3m* rhombohedral structural type. The absence of additional diffraction peaks allows one to conclude that samples are single-phase. The diffraction peaks’ position and intensity are in good agreement with the XRD data of the Inorganic Crystal Structure Database for the initial CuCrS_2_ matrix (card No. 100594, denoted as “ICSD” in Figure 1) [35]. This fact indicates that CuCr_0.99_Ln_0.01_S_2_ solid solutions and the initial matrix are isostructural. The calculated lattice parameters of CuCr_0.99_Ln_0.01_S_2_ are close to those of the CuCrS_2_ matrix and lie within the range of 3.47–3.48 and 18.68–18.70 Å for *a* and *c* parameters, respectively (Table 1). Thus, one can conclude that cationic substitution of the chromium by lanthanide atoms in the CuCrS_2_ matrix does not significantly affect the crystal structure of the matrix. The slight decrease in the unit cell volume is due to the lanthanide ionic radii contraction as the atomic number Z increases from 59 (Pr) to 65 (Tb).

### 3.2. X-ray Photoelectron Spectroscopy (XPS)

**Figure 2:**
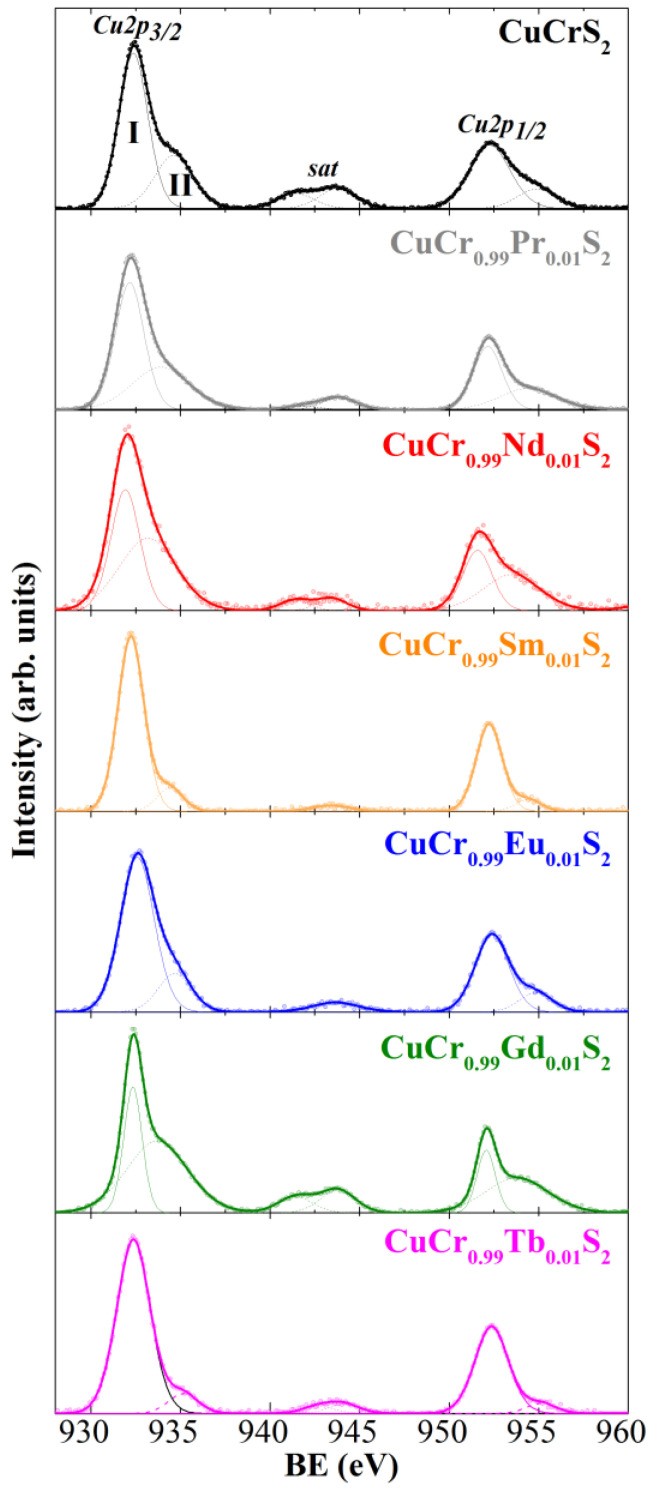
X-ray photoelectron Cu2p region for CuCrS_2_ and CuCr_0.99_Ln_0.01_S_2_ (Ln = Pr–Tb).

The XPS Cu2p lines of the initial CuCrS_2_ matrix and CuCr_0.99_Ln_0.01_S_2_ (Ln = Pr–Tb) solid solutions are shown in Figure 2. Table 2 lists the measured experimental binding energy (BE) values of XPS lines for the samples studied. The Cu2p line has a complicated structure. The Cu2p region exhibits a superposition of two intense main peaks, corresponding to 2p_3/2_ and 2p_1/2_ (denoted as Cu2p_3/2_ and Cu2p_1/2_ in Figure 2, respectively) levels accompanied by satellites (“sat” in Figure 2). The presence of the satellite lines in the Cu2p region is due to the charge-transfer process and is characteristic for compounds of Cu^2+^ [36]. In addition, as can be seen from Figure 2, the main Cu2p peaks are the superposition of two components arising from different types of copper atoms (I and II in Figure 2). The low-energy peaks I with a BE of 932.2–932.6 eV corresponded to the Cu^+^ state (Cu_2_S (BE ≈ 932.3 eV), Cu_2_O (BE ≈ 932.4 eV), CuCl (BE ≈ 932.3 eV) [37,38,39]). The high-energy peaks II of Cu2p_3/2_ lines (933.2–935.2 eV) corresponded to Cu^2+^ (CuO (BE ≈ 933.7 eV), CuCl_2_ (BE ≈ 933.7 eV), CuSO_4_(BE ≈ 935.4 eV) [37,38,39]) in the damaged and defective surface layers [16,40]. This fact is in good agreement with previously reported data for the initial CuCrS_2_ matrix and CuCrS_2_-based solid solutions [16,25,41]. For instance, the previously reported X-ray emission spectroscopy (XES) study considered the copper ions in CuCrS_2_-matrix as Cu^+^ [25,41]. The XES data indicated the absence of Cu^2+^ states in the bulk for CuCrS_2_-based solid solutions. This fact was additionally approved by magnetic susceptibility measurements [19,41,42]. Thus, one can conclude that the oxidation state of copper atoms in the composition of CuCr_0.99_Ln_0.01_S_2_ (Ln = Pr–Tb) solid solutions is Cu^+^.

**Figure 3:**
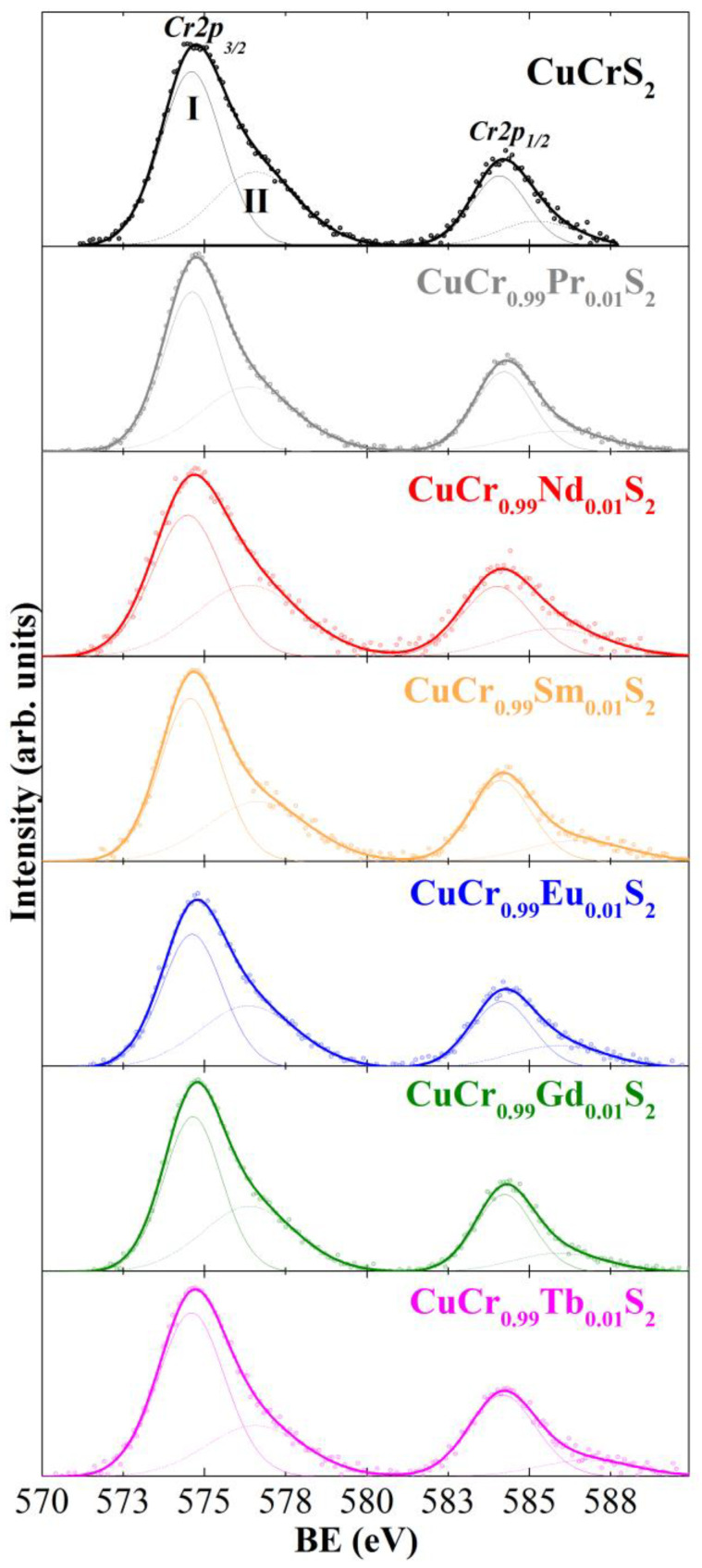
X-ray photoelectron Cr2p region for CuCrS_2_ and CuCr_0.99_Ln_0.01_S_2_ (Ln = Pr–Tb).

Figure 3 plots the Cr2p lines of the samples studied. The Cr2p region exhibits two intense peaks (BE of ~575 and ~584 eV) arising due to the spin-orbit coupling of Cr2p_1/2_ and Cr2p_3/2_ levels. The Cr2p_1/2_ and Cr2p_3/2_ lines could be represented as a superposition of two components (denoted as I and II in Figure 3). The low-energy peaks I with a BE of 574.6–574.7 eV are attributed to the Cr^3+^ state in the composition of chromium chalcogenides (Cr_2_S_3_ (BE ≈ 575.2 eV), CuCrSe_2_ (BE ≈ 574.7 eV), CuCr_2_Se_4_ (BE ≈ 574.5 eV) [37,38,39]). The high-energy peaks II with a BE of ~576.6 eV could correspond to the oxygen-containing compounds of Cr^3+^ in the near-surface layers of CuCr_0.99_Ln_0.01_S_2_ (Cr_2_O_3_ (BE ≈ 576.5 eV), CuCrO_2_ (BE ≈ 576.0 eV) [37,38,39]). Note that the obtained results are in accordance with previously reported data concerning the charge distribution in the initial CuCrS_2_ matrix and CuCrS_2_-based solid solutions [16,25,41]. Thus, the oxidation state of chromium atoms in CuCr_0.99_Ln_0.01_S_2_ is considered as Cr^3+^.

**Figure 4:**
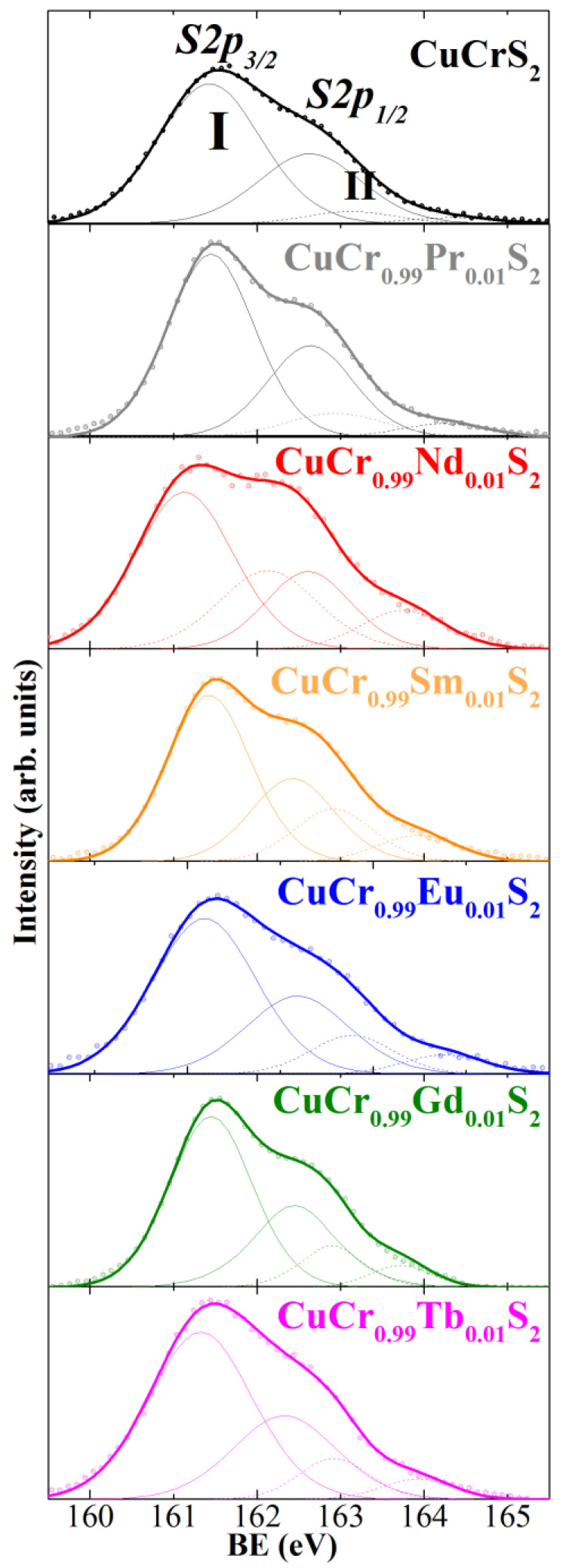
X-ray photoelectron S2p region for CuCrS_2_ and CuCr_0.99_Ln_0.01_S_2_ (Ln = Pr–Tb).

The S2p region of the initial CuCrS_2_ matrix and lanthanide-doped solid solutions is shown in Figure 4. The S2p line is unresolved peak arising due to spin-orbit coupling of S2p levels (denoted as S2p_1/2_- and S2p_3/2_ in Figure 4, respectively). The S2p region exhibited two sets of lines. The first one (denoted as I in Figure 4) with BE of 161.1–161.5 eV arises from the sulfur atoms in the composition of the samples studied and corresponds to S^2−^ state. The measured BE values is typical for transition metal sulfides (Cu_2_S (BE ≈ 161.8 eV), CuFeS_2_ (BE ≈ 161.5 eV), TiS_2_ (BE ≈ 160.9 eV) [36,37,38,39]). The high-energy component II with BE of ~163.0 eV arises from the sulfur atoms of polysulfide groups and elemental sulfur in the defective near-surface layers on the sample studied. However, the presence of the additional sulfur species on the sample surface is typical for the natural synthetic sulfides, including CuCrS_2_ [16,40,43,44]. Thus, the oxidation state of sulfur atoms in CuCr_0.99_Ln_0.01_S_2_ is considered as S^2−^.

**Figure 5:**
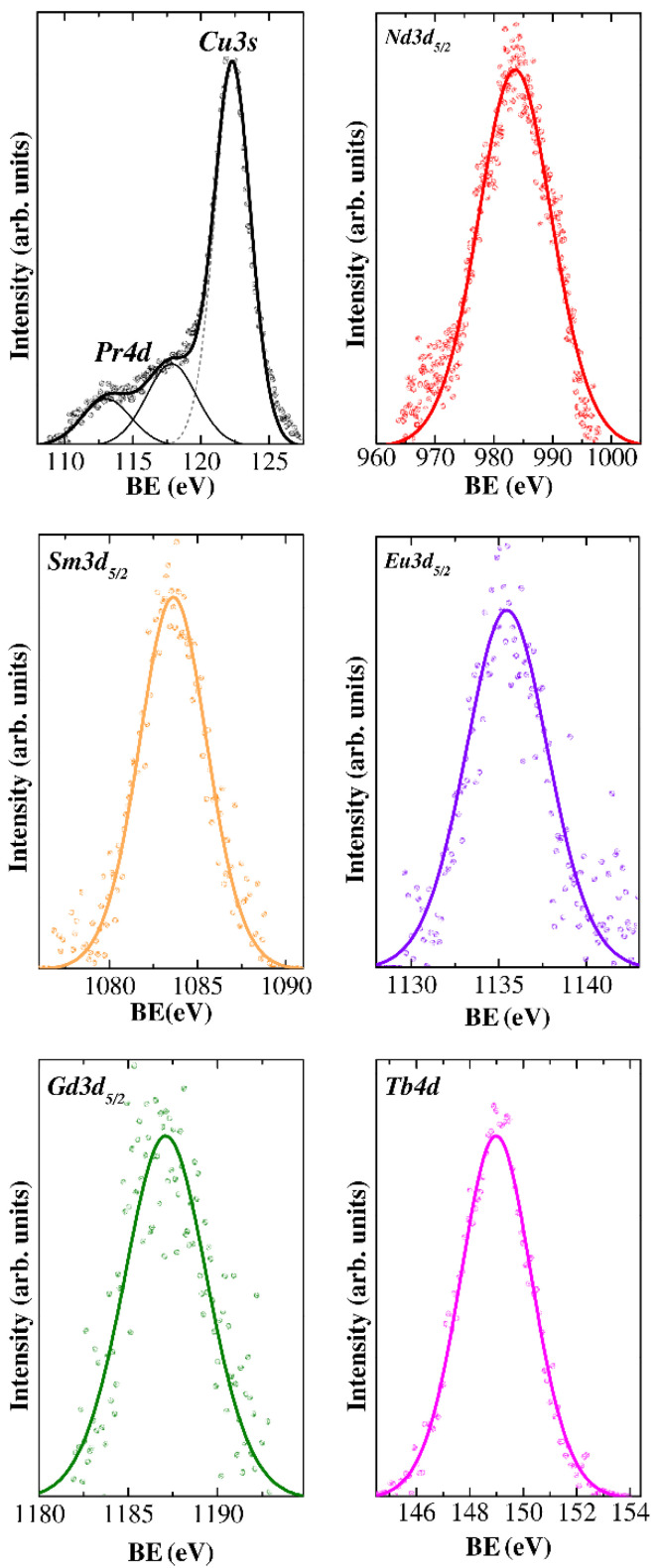
X-ray photoelectron lines of lanthanides for CuCr_0.99_Ln_0.01_S_2_ (Ln = Pr–Tb) solid solutions.

The study of the lanthanide oxidation state in CuCr_0.99_Ln_0.01_S_2_ solid solutions is of special interest due to the fact that contribution of the Ln4*f* level is assumed to affect the electronic structure and, thereby, the physical properties of the compounds studied [25]. The XPS lines of Pr, Nd, Sm, Eu, Gd and Tb in CuCr_0.99_Ln_0.01_S_2_ are shown in Figure 5. As can be seen in Figure 5 the Pr4d line lies in the same energy region as the Cu3s line. However, the deconvolution of the experimental data allowed one to measure the BE value of the Pr4d line as 117.9 eV (Table 2). The measured BE is close to one for Pr_2_O_3_ (BE ≈ 117.5 eV) and, thereby, corresponds to Pr^3+^ [45]. The Ln3d_5/2_ region (Ln = Nd, Sm, Eu, Gd) exhibited an intense single peak (Figure 5). The BE values of corresponding lines are listed in Table 2. The measured BE values are in good agreement with those for the lanthanide oxides Ln_2_O_3_ (Nd—983.1 eV, Sm—1083.5 eV, Eu—1135.3 eV and Gd—1186.8 eV), and correspond to the Ln^3+^ oxidation state [46,47,48]. In the case of terbium, the Tb3d line lies in the same energy region as the carbon Auger KVV line; hence, the Tb4d line was recorded. The BE value of 149.2 eV for the Tb4d line in CuCr_0.99_Tb_0.01_S_2_ corresponds to Tb^3+^ (Tb_2_O_3_ (BE ≈ 149.1 eV) [49]). Thus, the data analyzed allowed one to conclude that the oxidation state of lanthanide atoms in CuCr_0.99_Ln_0.01_S_2_ is Ln^3+^. The absence of significant chemical shifts (≥0.2 eV, taking into account recoil effects and the crystal lattice vibrations) of the measured copper, chromium and sulfur XPS lines indicated electron density preservation during both the cationic substitution process and variation of the lanthanide element.

**Figure 6:**
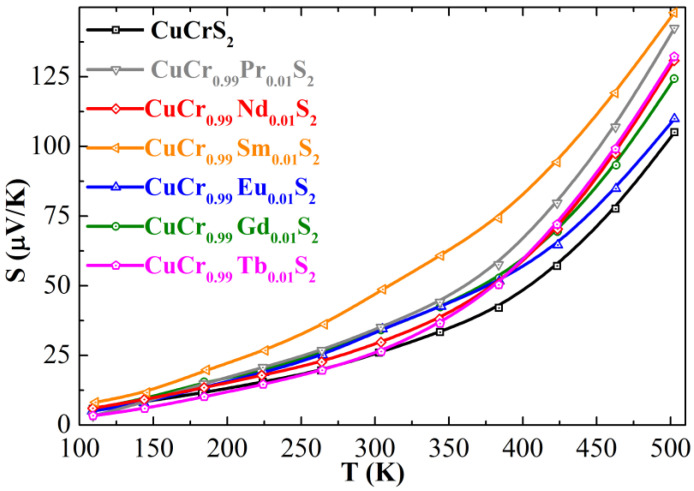
Seebeck coefficient temperature dependencies of CuCrS_2_ and CuCr_0.99_Ln_0.01_S_2_ (Ln = Pr–Tb) solid solutions.

Taking into account the obtained data on the charge distribution on the metal and sulfur atoms in CuCr_0.99_Ln_0.01_S_2_, one can conclude that cationic substitution occurs via the isovalent mechanism Cr^3+^→Ln^3+^, which is in good agreement with previously reported studies of CuCr_0.99_Ln_0.01_S_2_ magnetic properties [19,42]. For instance, the experimental value of the effective magnetic moment for CuCr_0.99_Ln_0.01_S_2_ correlated with the theoretical values that consider Cr^3+^→Ln^3+^ isovalent substitution [42]. It should be noted that magnetic susceptibility is a macroscopic property of chemical compounds, whereas XPS spectroscopy allows one to analyze the oxidation state of elements directly [32,39,41]. Thus, the lanthanide atoms should exhibit neither donor nor acceptor properties with respect to the initial CuCrS_2_ matrix. However, the measured Seebeck coefficient temperature dependencies of the compounds studied indicated that cationic substitution causes the enhancement in the Seebeck coefficient values for CuCr_0.99_Ln_0.01_S_2_ in comparison with those for the initial matrix (Figure 6). The largest Seebeck coefficient values of 142 and 148 µV/K were observed at 500 K for CuCr_0.99_Sm_0.01_S_2_ and CuCr_0.99_Pr_0.01_S_2_, respectively. Note that the obtained values are 1.4 times greater in comparison with those for the initial matrix (105 µV/K). An analysis of Equation (1) allows one to conclude that the observed enhancement from could, on the one hand, be associated with the charge carrier properties alteration, and on the other hand, with the electronic structure reconfiguration after the cationic substitution. As mentioned above, due to the fact that cationic substitution in CuCr_0.99_Ln_0.01_S_2_ occurs via an isovalent mechanism, one can consider that the charge carrier concentration should not affect the Seebeck coefficient value. However, the carrier mobility decrease should lead to the Seebeck coefficient enhancement. Since one of the most significant factors affecting the carrier mobility is their scattering, the greatest effect should be observed for lanthanide atoms bearing the highest effective magnetic moment [26,30,31,50]. Nevertheless, despite the fact that, in the studied series of lanthanides (Pr to Tb), gadolinium has the largest magnetic moment value, the most significant increase in Seebeck coefficient value is observed for the samarium-doped solid solution (S = 148 µV/K). Thus, one can conclude at least as a first approximation, that the observed behavior of thermoelectric properties for CuCr_0.99_Ln_0.01_S_2_ (Ln = Pr–Tb) corresponds to the electronic structure reconfiguration (i.e., the effective DOS in the valence band top and conduction band bottom).

## 4. Conclusions

The charge distribution in CuCr_0.99_Ln_0.01_S_2_ (Ln = Pr–Tb) thermoelectric materials was studied. It was shown that the copper atoms in the composition of CuCr_0.99_Ln_0.01_S_2_ are found to be in the Cu^+^ state. The lanthanide and chromium atoms were found to be in the trivalent state. The sulfur atoms were found to be divalent. Thus, cationic substitution of the initial CuCrS_2_ matrix does not significantly affect the distribution of electron density in CuCr_0.99_Ln_0.01_S_2_ solid solutions. The cationic substitution was found to occur via the isovalent mechanism of Cr^3+^ to Ln^3+^. Contributions arising from the elemental sulfur, the polysulfide groups, the copper and chromium oxygen-containing compounds in the near-surface layers of CuCr_0.99_Ln_0.01_S_2_ were observed. The isovalent mechanism of the cationic substitution allows one to exclude from consideration the influence of the lanthanide donor/acceptor properties as a promotor of the Seebeck coefficient increase of CuCr_0.99_Ln_0.01_S_2_ (Ln = Pr–Tb). The possible improvement in thermoelectric properties could correspond to changes in the electronic structure (i.e., valence band top and conduction band bottom) due to the presence of lanthanide 4*f*-states after the cationic substitution of chromium atoms in the initial CuCrS_2_ matrix with lanthanide ions.

## Figures and Tables

**Table 1:** Lattice parameters for the initial CuCrS_2_ matrix and CuCr_0.99_Ln_0.01_S_2_ (Ln = Pr, Nd, Sm, Eu, Gd, Tb) solid solutions.

	*a*, Å	*c*, Å
CuCrS_2_	3.48 (3)	18.68 (9)
CuCr_0.99_Pr_0.01_S_2_	3.48 (2)	18.70 (1)
CuCr_0.99_Nd_0.01_S_2_	3.47 (9)	18.68 (8)
CuCr_0.99_Sm_0.01_S_2_	3.47 (9)	18.68 (6)
CuCr_0.99_Eu_0.01_S_2_	3.47 (9)	18.68 (5)
CuCr_0.99_Gd_0.01_S_2_	3.48 (0)	18.69 (6)
CuCr_0.99_Tb_0.01_S_2_	3.48 (0)	18.69 (7)

**Table 2:** Binding energy values of Cu2p_3/2_, Cr2p_3/2_, S2p_3/2_, Ln3d_5/2_ (Ln = Nd–Gd) and Ln4d lines (Ln = Pr, Tb) for CuCr_0.99_Ln_0.01_S_2_.

BE ± 0.2 eV	Cu2p_3/2_	Cr2p_3/2_	S2p_3/2_	Ln3d_5/2_
CuCrS_2_	932.4	574.6	161.5	–
	934.6	576.6	163.1	
CuCr_0.99_Pr_0.01_S_2_	932.2	574.6	161.5	117.9 (Pr4d)
	933.9	576.4	162.9	
CuCr_0.99_Nd_0.01_S_2_	932.2	574.5	161.1	988.6
	933.2	576.4	162.6	
CuCr_0.99_Sm_0.01_S_2_	932.2	574.6	161.4	1083.6
	934.4	576.7	162.9	
CuCr_0.99_Eu_0.01_S_2_	932.6	574.6	161.2	1135.5
	934.7	576.4	162.8	
CuCr_0.99_Gd_0.01_S_2_	932.3	574.7	161.5	1187.1
	933.7	576.4	162.9	
CuCr_0.99_Tb_0.01_S_2_	932.4	574.6	161.3	152.7 (Tb4d)
	935.2	576.6	162.9	

## Data Availability

Not applicable.
